# Crystal structures of PCNA mutant proteins defective in gene silencing suggest a novel interaction site on the front face of the PCNA ring

**DOI:** 10.1371/journal.pone.0193333

**Published:** 2018-03-02

**Authors:** Christine M. Kondratick, Jacob M. Litman, Kurt V. Shaffer, M. Todd Washington, Lynne M. Dieckman

**Affiliations:** 1 Department of Biochemistry, University of Iowa College of Medicine, Iowa City, IA, United States of America; 2 Department of Chemistry, Creighton University, Omaha, NE, United States of America; Saint Louis University, UNITED STATES

## Abstract

Proliferating cell nuclear antigen (PCNA), a homotrimeric protein, is the eukaryotic sliding clamp that functions as a processivity factor for polymerases during DNA replication. Chromatin association factor 1 (CAF-1) is a heterotrimeric histone chaperone protein that is required for coupling chromatin assembly with DNA replication in eukaryotes. CAF-1 association with replicating DNA, and the targeting of newly synthesized histones to sites of DNA replication and repair requires its interaction with PCNA. Genetic studies have identified three mutant forms of PCNA in yeast that cause defects in gene silencing and exhibit altered association of CAF-1 to chromatin *in vivo*, as well as inhibit binding to CAF-1 *in vitro*. Three of these mutant forms of PCNA, encoded by the *pol30-6*, *pol30-8*, and the *pol30-79* alleles, direct the synthesis of PCNA proteins with the amino acid substitutions D41A/D42A, R61A/D63A, and L126A/I128A, respectively. Interestingly, these double alanine substitutions are located far away from each other within the PCNA protein. To understand the structural basis of the interaction between PCNA and CAF-1 and how disruption of this interaction leads to reduced gene silencing, we determined the X-ray crystal structures of each of these mutant PCNA proteins. All three of the substitutions caused disruptions of a surface cavity on the front face of the PCNA ring, which is formed in part by three loops comprised of residues 21–24, 41–44, and 118–134. We suggest that this cavity is a novel binding pocket required for the interaction between PCNA and CAF-1, and that this region in PCNA also represents a potential binding site for other PCNA-binding proteins.

## 1. Introduction

Efficient replication and repair of the eukaryotic genome is essential for the maintenance of genomic and epigenetic integrity. During the S phase of the cell cycle, newly synthesized DNA is assembled into chromatin in a rapid and precise manner. The basic unit of chromatin is the nucleosome, which consists of approximately 147 base pairs of DNA wrapped around an octamer of histone proteins, containing two copies each of the histones H2A, H2B, H3, and H4 [1]. These histone proteins are subject to extensive covalent modifications that regulate chromatin structure and gene expression [2], and the identity of a cell is maintained partially through the preservation of these posttranslational modifications on chromatin during assembly. The process of nucleosome assembly immediately following DNA replication, involving the incorporation of both parental and newly synthesized histones, is called replication-coupled nucleosome assembly. During this process, newly synthesized histones H3 and H4 are deposited on DNA as tetramers by a histone chaperone protein called chromatin assembly factor-1 (CAF-1) [3]. This is followed by deposition of two sets of H2A-H2B dimers [4] onto the tetramer to complete the nucleosome [5].

CAF-1 is a heterotrimeric protein that is conserved in eukaryotes from yeast to humans and is required for heterochromatin formation [6–9] and coupling chromatin assembly with DNA replication [10–12]. In yeast, CAF-1 consists of the subunits Cac1, Cac2, and Cac3. Inactivation of CAF-1 in cells increases the accumulation of chromosomal re-arrangements and activates DNA damage checkpoints [13], and deletion of any one of the CAF-1 subunits results in partial loss of telomeric silencing and increased sensitivity to UV irradiation [14]. In addition to replication-coupled nucleosome assembly, studies suggest that CAF-1 facilitates chromatin restoration following nucleotide excision repair [15–17], double-strand break repair [18,19], and mismatch repair [20–22].

CAF-1 association with replicating DNA, and the targeting of newly synthesized histones to sites of DNA replication and repair requires its interaction with proliferating cell nuclear antigen (PCNA) [23–25], the eukaryotic sliding clamp that acts as a processivity factor for polymerases during DNA synthesis. Disruption of the CAF-1-PCNA interaction in yeast inhibits CAF-1-mediated heterochromatin silencing and chromatin assembly during DNA replication and repair [8,24–27]. Therefore, the direct interaction between CAF-1 and PCNA is critical for coupling DNA replication to nucleosome assembly; however, the precise role of this interaction in this process is not well understood.

**Fig 1:**
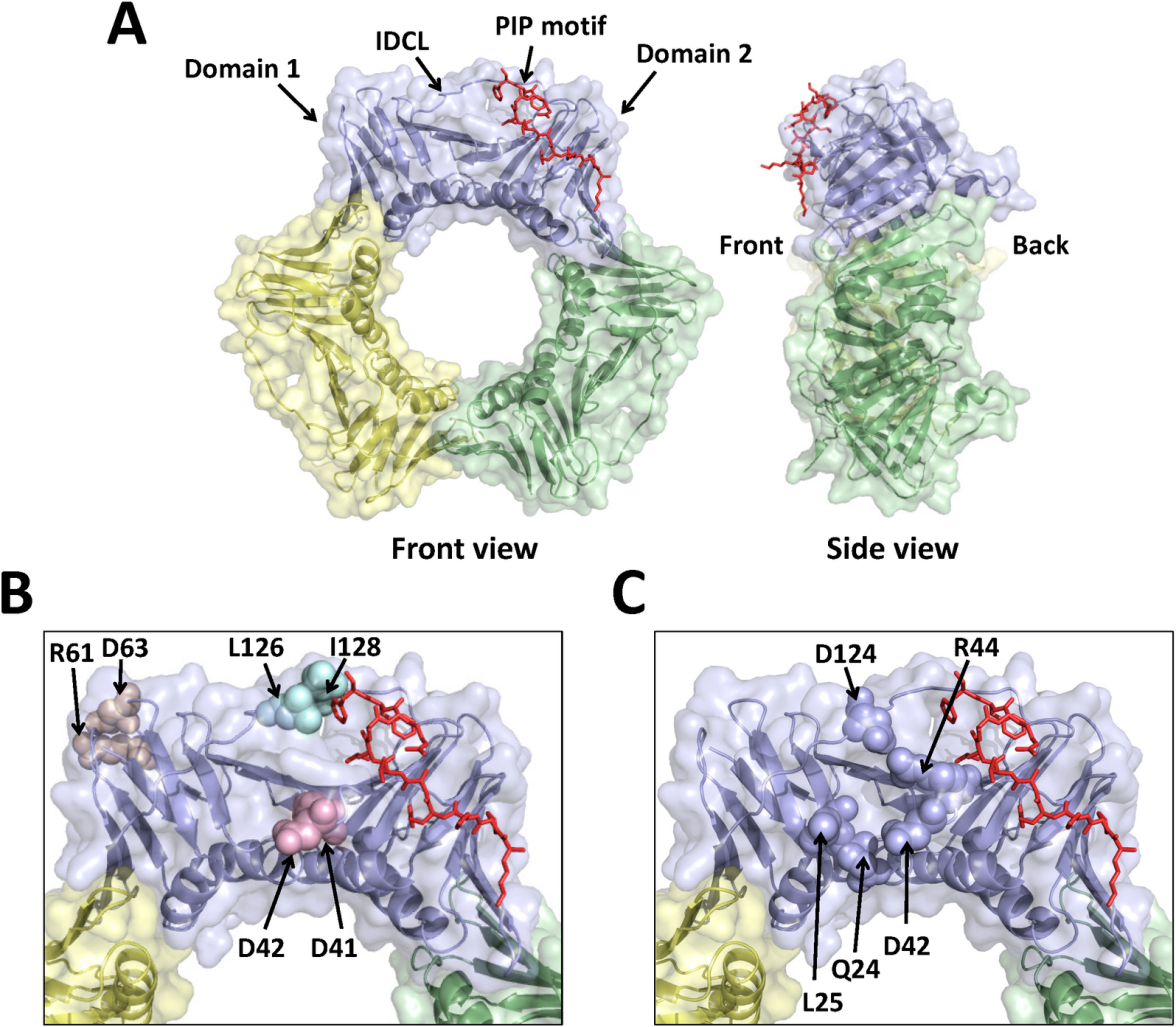
Location of the three double alanine substitutions in PCNA. (A) Front and side views of the wild-type PCNA trimer (PDB entry 2OD8.pdb). The three subunits of PCNA are colored light blue, pale green, and pale yellow. The bound PIP motif is shown in the stick representation and colored red. (B) Close-up view of one subunit of PCNA with the location of each substituted amino acid residue shown in the sphere representation. The D41A/D42A, R61A/D63A, and L126A/I128A substitutions are colored light pink, wheat, and pale cyan, respectively. (C) Close-up view of one subunit of PCNA with the location of the residues comprising the surface cavity shown in the sphere representation.

PCNA is a ring-shaped homotrimeric protein, with each subunit consisting of two domains connected by a flexible linker called the inter-domain connecting loop (IDCL) (Fig 1A). During most DNA replication-linked processes, PCNA encircles double-stranded DNA and recruits many of the proteins involved in these processes to sites of DNA synthesis. PCNA recruits more than 50 different proteins involved in DNA replication, DNA repair, transcription, homologous recombination, chromatin assembly and remodeling, and cell cycle regulation [28–34]. Recruitment of PCNA-interacting proteins to replication forks is generally mediated by a conserved sequence in these proteins called a PCNA-interacting protein (PIP) motif that interacts with PCNA in a region between the two domains on a single subunit near the IDCL. The interaction between CAF-1 and PCNA is presumed to be mediated through a PIP motif on the largest subunit of CAF-1 (p150 in humans [24] and Cac1 in yeast [25]) and the PIP-interacting region on PCNA [24,29]; however, no detailed structural analysis has been carried out to confirm this hypothesis.

Several PCNA mutant proteins have been identified in yeast that exhibit altered association of CAF-1 to chromatin as well as gene silencing defects. Our studies focused on three of these mutant forms of PCNA–those with the amino acid substitutions D41A/D42A, R61A/D63A, and L126A/I128A, encoded by the *pol30-6*, *pol30-8*, and the *pol30-79* alleles, respectively (Fig 1B). These three mutant proteins demonstrated normal PCNA localization to replicating DNA but showed decreased binding to CAF-1 *in vitro* [25]. To understand the structural basis of the interaction between PCNA and CAF-1 and how disruption of this interaction leads to reduced gene silencing, we determined the X-ray crystal structure of each of these three mutant PCNA proteins. All three of the PCNA mutant proteins have changes in a surface cavity formed by three loops comprised of residues 21–24, 41–44, and 118–134. This cavity is on the front face of the ring and is approximately 10 Å away from the PIP-interacting region (Fig 1C). This surface cavity in PCNA may represent a novel binding pocket required for interacting with CAF-1 as well as a potential binding site for interacting with other PCNA-binding proteins.

## 2. Materials and methods

### 2.1. Protein expression and purification

Wildtype PCNA, mutant D41A/D42A PCNA, mutant R61A/D63A PCNA, and mutant L126A/I128A PCNA from *Saccharomyces cerevisiae* were overexpressed as N-terminally His_6_- tagged proteins in *E*. *coli* and were purified as described previously [35].

### 2.2. Native gel electrophoresis

Wild-type PCNA and the PCNA mutant proteins (D41A/D42A, R61A/D63A, L126A/I128A, and G178S) were diluted to various concentrations (0.1, 0.5, 1.0 mg/ml) and incubated in 10 mM Tris-Cl, pH 7.6, 150 mM NaCl, 2.5% Ficoll-400, 10 mM EDTA, 0.03% xylene cyanol, and 0.03% Orange G for 5 min. Proteins were then run on a precast 4–20% gradient, nondenaturing Tris-Glycine gel (Bio-Rad) at 4°C at a current of 25 mA using 25 mM Tris, pH 8.3, and 0.2 M glycine running buffer. Protein bands were visualized using Coomassie blue staining.

### 2.3. Crystallization

Crystallization of all mutant PCNA proteins were performed using the hanging-drop method at 18°C. The D41A/D42A PCNA protein was concentrated to 28 mg/ml for crystallization and protein crystals were formed in 0.1 M sodium citrate, pH 5.43, 0.529 M ammonium sulfate, and 0.729 M lithium sulfate. The R61A/D63A protein crystalized at a concentration of 20 mg/ml in 0.1 M sodium citrate, pH 5.57, 0.671 M ammonium sulfate, and 0.994 M lithium sulfate. The L126A/I128A protein crystalized at a concentration of 21 mg/ml in 0.1 M sodium cacodylate, pH 6.5, 0.2 M magnesium chloride, and 20% w/v PEG1000.

### 2.4 Data collection and structural determination

The D41A/D42A crystals were looped and immediately flash cooled in liquid nitrogen prior to data collection using a Rigaku rotating anode X-ray source. The R61A/D63A and L126A/I128A protein crystals were soaked in mother liquor containing 20% (w/v) glycerol prior to flash cooling with liquid nitrogen. The data collection for these mutant proteins was carried out at the 4.2.2 synchrotron beamline at the Advanced Light Source in the Ernest Orlando Lawrence Berkeley National Laboratory. The D41A/D42A and R61A/D63A crystal diffraction data was processed and scaled using d*TREK [36] and both mutant proteins were determined to be in the P213 space group. For the L126A/I128A mutant crystal, the diffraction data was processed with XDS [37] and the space group was determined to be R3. The L128A/I128A mutant protein was phased using molecular replacement using wild-type PCNA (PDB 1PLQ) with PHASER [38]. Since the D41A/D42A and the R61A/D63A mutant proteins were nearly isomorphic with the wild-type protein, ten rounds of rigid body refinement using the wild-type structure were sufficient to place the mutant proteins in the unit cell. Model building and refinement were done using Coot [39], REFMAC5 [40], PHENIX [41], and FFX [42,43].

## 3. Results

### 3.1. Stability of the PCNA mutant proteins

Fig 1B shows the location of the three double alanine substitutions within the PCNA trimer. The D41A/D42A substitutions are located on the front of the PCNA ring in loop D (residues 41 to 44), which is approximately 10 Å away from the canonical PIP-interacting region. The R61A/D63A substitutions are located on the side of PCNA adjacent to and within loop F (residues 62 to 65), which is over 30 Å away from the PIP-interacting region. The L126A/L128A substitutions are located within the IDCL (residues 118 to 134), which is directly adjacent to the PIP-interacting region. With the exception of D42, which is a serine in plants and animals, these residues are very highly conserved among eukaryotes.

**Fig 2:**
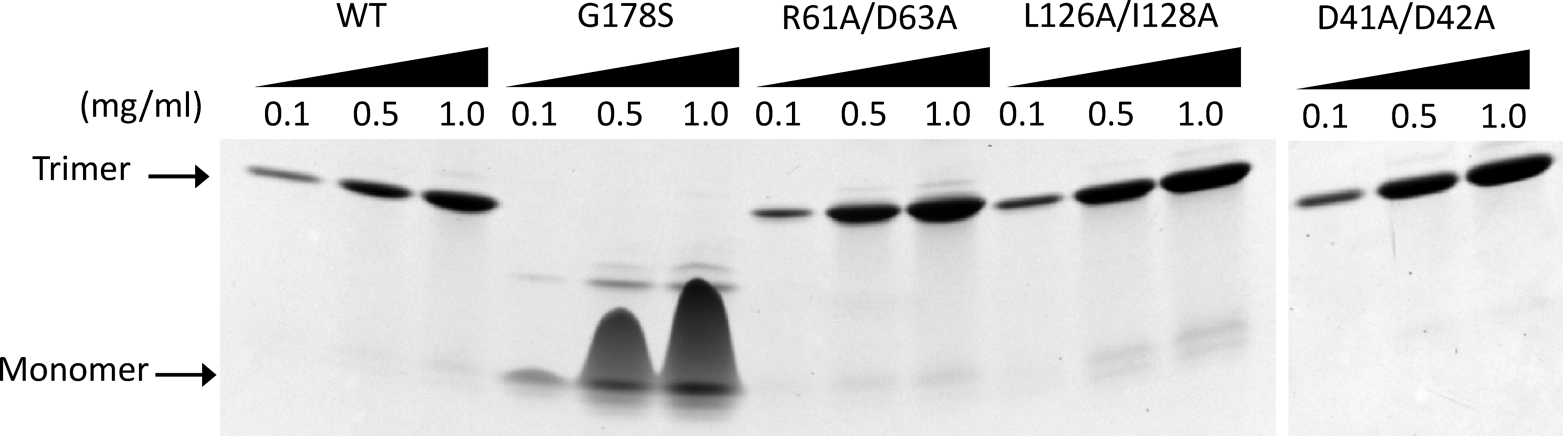
Native gel electrophoresis of wild-type and mutant PCNA proteins. Solutions of the wild-type PCNA protein, the G178S mutant PCNA protein, the R61A/D63A mutant PCNA protein, the L126A/I128A mutant PCNA protein, and the D41A/D42A mutant PCNA protein (0.1–1.0 mg/ml) were run on a non-denaturing polyacrylamide gradient gel (4–20%) and stained with Coomassie blue. The positions of the PCNA monomer and PCNA trimer are indicated.

Previous studies of various loss-of-function PCNA mutant proteins have revealed that the destabilization of the PCNA quaternary structure is a common means by which mutations result in altered function *in vivo* [44–46]. To rule out the possibility that the *in vivo* effects of the three amino acid substitutions studied here are not due to the formation of aberrant protein structures, we examined the stability of the mutant PCNA proteins. We used native polyacrylamide gel electrophoresis (PAGE) to examine the oligomeric state of each mutant PCNA protein at various concentrations (Fig 2). These oligomeric states were compared to those formed by wild-type PCNA and a mutant form of PCNA that is unable to form stable trimers (G178S) [44]. All three mutant PCNA proteins form stable trimers at the concentrations tested (0.1–1.0 mg/mL), which approximate the concentration of PCNA in the nucleus (3 mg/ml) [47]. These results show that disruption of the CAF-1 interaction observed in these mutant proteins is not due to a loss of quaternary structure and thus suggest that the amino acid substitutions are genuine separation-of-function mutations.

### 3.2. Structure of the D41A/D42A PCNA mutant protein

**Fig 3:**
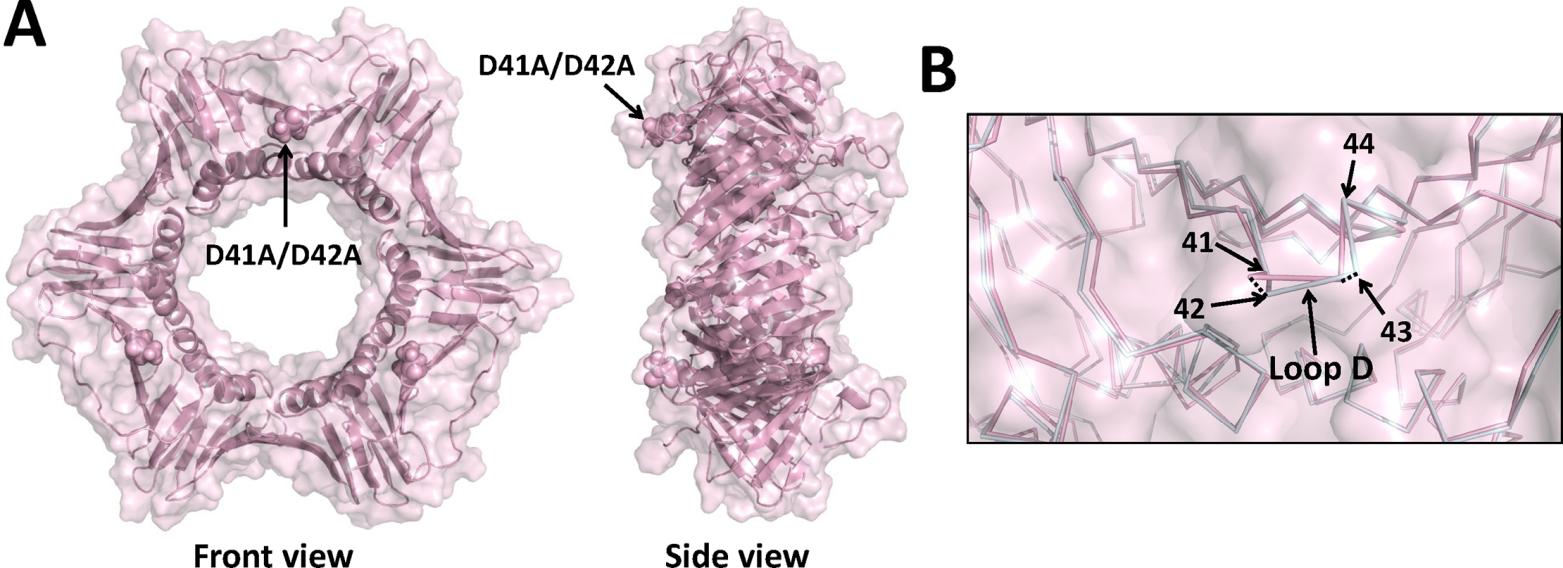
X-ray crystal structure of the D41A/D42A mutant PCNA protein. (A) Front and side views of the D41A/D42A mutant PCNA protein. All three subunits are colored light pink, and the locations of the substituted amino acid residues are shown in the sphere representation. (B) Close up of an overlay of the wild-type PCNA protein (light blue) and the D41A/D42A mutant PCNA protein (light pink) are shown in the ribbon representation (RMSD of 0.529 Å). The positions of the α carbons of residues 41 to 44 are indicated.

The X-ray crystal structure of the D41A/D42A mutant protein was determined to a resolution of 3.05 Å (Table 1). The D41A/D42A substituitions are located on the front face of PCNA in loop D (residues 41–44) between β strands C_1_ and D_1_. These β strands lie beneath the IDCL (residues 118–134) in domain 1 of PCNA. The global structure of the mutant PCNA trimer is very similar to the structure of the wild-type PCNA trimer (Fig 3A). There are, however, a few notable local structural changes. Compared to the wild-type structure, there are significant backbone changes in the structure of the D41A/D42A mutant PCNA protein in loop D. The α carbon of residue 42 has moved by 1.2 Å relative to its position in the wild-type PCNA structure, and the α carbon of residue 43 has moved by 1.0 Å (Fig 3B, Table 2). These backbone changes in loop D and the substitutions of the shorter alanine sidechains at residues 41 and 42 are the only significant changes in the structure of the mutant PCNA protein compared to the structure of the wild-type PCNA protein.

**Table 1:** Data collection and refinement statistics.

Data set	D41A/D42A	R61A/D63A	L126A/I128A
PDB code	5V7K	5V7L	5V7M
Space group	P213	P213	R3
Unit-cell parameters			
*a* (Å)	122.32	123.18	81.72
*b* (Å)	122.32	123.18	81.72
*c* (Å)	122.32	123.18	88.99
α (°)	90	90	90
β (°)	90	90	90
γ (°)	90	90	120
Resolution range (Å)	14.83–3.05	43.55–3.2	55.39–1.93
Total No. of reflections^a^	128106(18952)	233446(34305)	92277(6060)
No. of unique reflections^a^	11773(1716)	10563(1514)	16678(157)
Completeness (%)^a^	99.1(100.0)	100(100)	100(100)
Multiplicity^a^	10.9(11.0)	22.1(22.7)	5.5(5.3)
Mean *I*/σ (*I*)^a^	16.1(2.0)	24.9(2.0)	13.1(1.2)
*R*_merge_ (%)^a^	16.0(143.0)	11.5(174.4)	10.1(109.6)
R _pim_ (%)^a^	6.7(59.7)	3.5(53.0)	7.4(82.5)
*R*_work_ / *R*_free_ (%)	20.13/ 24.82	19.94/25.06	18.15/23.54
RMSD			
Bond lengths (Å)	0.019	0.004	0.012
Bond angles (°)	2.749	0.791	1.200
Ramachandran plot (%)			
Favoured	92.55	93.28	97.24
Allowed	7.45	6.72	2.76
Outliers	0.0	0.0	0.0
Rotamer outliers (%)	6.67	3.95	2.63

^a^Values in parentheses are for highest-resolution shell. The *I*/σ(*I*) is 1.2 at 1.93 Å, but the CC(1/2) is 0.507 which indicates significant information for the reflections at this resolution. The *I*/σ(*I*) is 2.1 at 2.05 Å.

**Table 2:** Backbone α-carbon displacement.

Residue	D41A/D42A	R61A/D63A	L126A/I128A
**Loop B**			
D21	0.9	1.2	1.2
C22	0.7	2.6	1.3
V23	0.7	0.8	1.4
Q24	0.4	0.9	1.0
L25	0.6	0.2	0.7
**Loop D**			
D41	0.6	0.3	1.5
D42	1.2	0.3	2.7
S43	1.0	0.5	2.5
R44	0.3	0.4	2.1
**Loop F**			
R61	0.5	0.5	0.5
C62	0.5	0.4	0.8
D63	1.0	0.8	1.6
**IDCL**			
A123	1.0	2.0	1.4
D124	0.4	1.1	2.5
F125	0.5	0.7	1.8
L126	0.6	0.5	1.8
K127	0.6	0.3	2.9
I128	0.9	0.4	2.6
E129	0.8	0.3	1.9

For comparison, the RMSD values between the wild-type and D41A/D42A structures, the wild-type and R61A/D63A structures, and the wild-type and L126A/I128A structures were 0.529 Å, 0.317 Å, and 0.835 Å, respectively.

### 3.3. Structure of the R61A/D63A PCNA mutant protein

**Fig 4:**
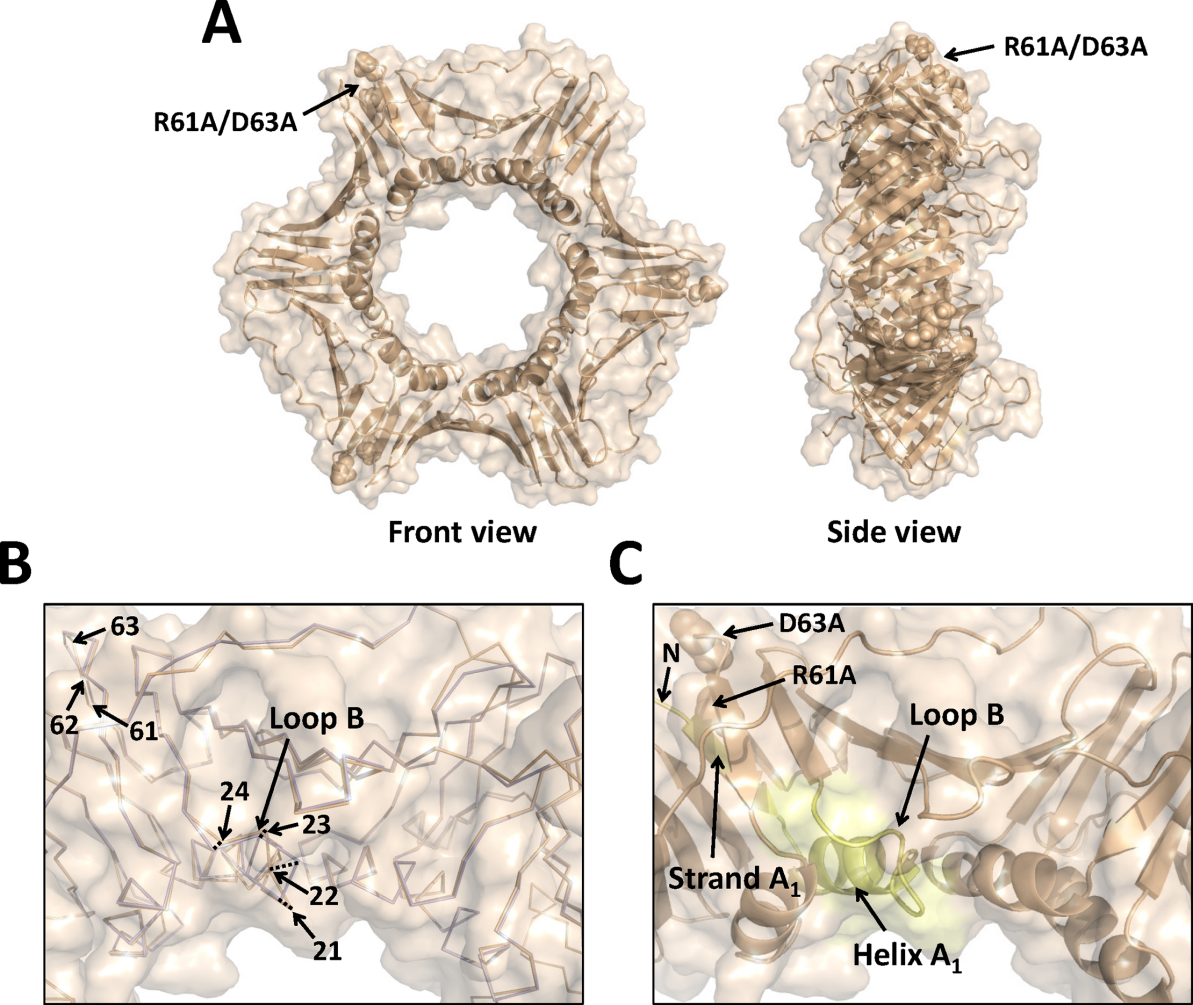
X-ray crystal structure of the R61A/D63A mutant PCNA protein. (A) Front and side views of the R61A/D63A mutant PCNA protein. All three subunits are colored wheat, and the locations of the substituted amino acid residues are shown in the sphere representation. (B) Close up of an overlay of the wild-type PCNA protein (light blue) and the R61A/D63A mutant PCNA protein (wheat) are shown in the ribbon representation (RMSD of 0.317 Å). The positions of the α carbons of residues 21 to 24 and of residues 61 to 63 are indicated. (C) Close up of the structure of the R61A/D63A mutant PCNA protein shown in the cartoon representation. The N-terminus, β strand A_1_, α helix A_1_, and loop B are colored pale yellow.

The X-ray crystal structure of the R61A/D63A mutant protein was determined to a resolution of 3.3 Å (Table 1). The R61A/D63A substitutions are located on the side of the PCNA ring and far from from the locations of the other two double alanine substitutions. The R61A substitution is located in β strand E_1_ and the D63A substitution is located in loop F (residues 62–65) in domain 1 of PCNA. Again, the global structure of the mutant PCNA trimer is very similar to the structure of the wild-type PCNA trimer (Fig 4A). There are local structural changes both at the sites of the amino acid substitutions and at a site more than 30 Å away from these substitutions. At the site of the substitutions, there are small backbone changes around residues 61 and 62. The α carbons of the substituted residues and the other residues in loop F have all moved by less than 1 Å relative to their positions in the wild-type PCNA structure (Fig 4B, Table 2). Although the substitutions at residues 61 and 63 only appear to cause minor structural changes near these positions, they produce significant changes in loop B (residues 21 to 24), a region of the PCNA monomer that is more than 30 Å away from the substitutions. Compared to the wild-type structure, there are significant backbone changes in the structure of the R61A/D63D mutant PCNA protein in loop B. The α carbon of residue 21 has moved by 1.2 Å relative to its position in the wild-type PCNA structure, and the α carbon of residue 22 has moved by 2.6 Å (Fig 4B, Table 2).

Although the precise reason for this induced, long-range structural change is unclear, it is likely mediated through β strand A_1_ (residues 2 to 6) and α helix A_1_ (residues 9 to 20). The substitutions in loop F are immediately adjacent to the N terminus of the protein. This could induce changes in β strand A_1_, which in turn induce changes in α helix A_1_, which in turn induce changes in loop F. In support of this notion, the α carbons on α helix A_1_ that is immediately upstream of loop B in the mutant protein structure have also moved by approximately 2 Å relative to their positions in the structure of the wild-type protein.

### 3.4. Structure of the L126A/I128A PCNA mutant protein

**Fig 5:**
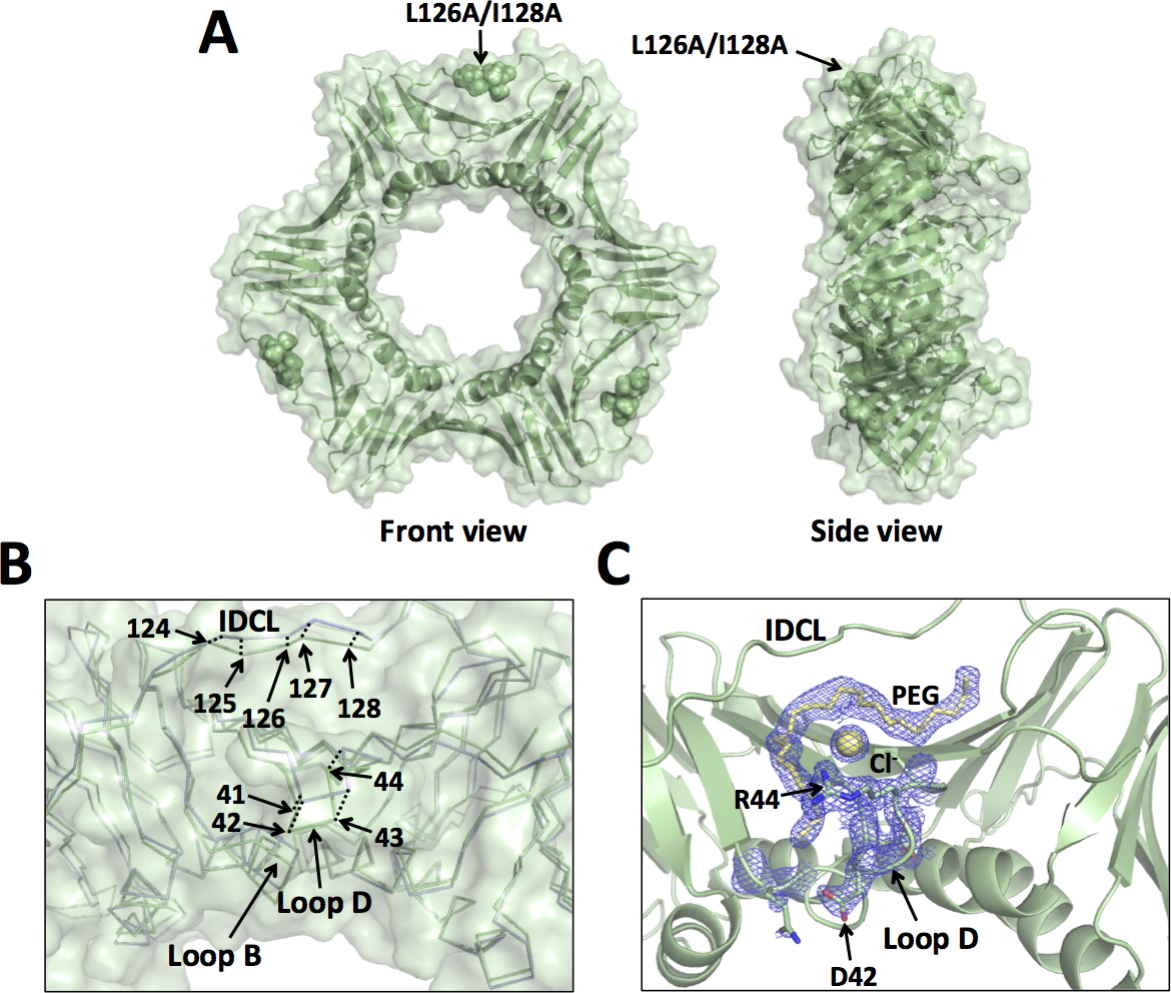
X-ray crystal structure of the L126/I128A mutant PCNA protein. (A) Front and side views of the L126A/I128A mutant PCNA protein. All three subunits are colored pale green, and the locations of the substituted amino acid residues are shown in the sphere representation. (B) Close up of an overlay of the wild-type PCNA protein (light blue) and the L126A/I128A mutant PCNA protein (pale green) are shown in the ribbon representation (RMSD of 0.835 Å). The positions of the α carbons of residues 41 to 44 and of residues 124 to 128 are indicated. (C) Close up of the structure of the L126A/I128A mutant PCNA protein shown in the cartoon representation. The side chains of D42 and R44 are shown in the stick representation. A portion of a PEG molecule and a chloride ion are shown in the stick and sphere representation, respectively. The 2Fo-Fc map contoured at 1 σ is shown.

The X-ray crystal structure of the L126A/I128A mutant PCNA protein was determined to a resolution of 1.93 Å (Table 1). The L126A/I128A residues are located within the IDCL of PCNA, which is directly above the canonical PIP-interacting region. The global structure of the mutant PCNA trimer is very similar to that of the wild-type PCNA trimer (Fig 5A). Multiple, local structural changes, however, are apparent. Compared to the wild-type structure, there are significant backbone changes in the structure of the L126A/I128A mutant PCNA in the IDCL. The α carbons of residues 123 to 129 have moved from 1.8 to 2.9 Å relative to their positions in the wild-type PCNA structure (Fig 5B, Table 2). Similarly, there are significant backbone changes in the structure of the mutant PCNA protein in loop B, which is more than 15 Å from the substitutions. The α carbons of residues 21, 22, and 23 have moved by 1.2, 1.3, and 1.4 Å relative to their positions in the wild-type PCNA structure (Fig 3B, Table 2). These structural changes in loops B are unlikely due to this mutant protein being crystalized in a different space group. This is because this loop is solvent exposed and are not located near any symmetry related PCNA molecules in either space group. Finally, there are significant backbone changes in the structure of the mutant PCNA protein in loop D, which is approximately 15 Å from the substitutions. The α carbons of residues 41 to 44 have moved from 1.5 to 2.7 Å relative to their positions in the wild-type PCNA structure (Fig 5B, Table 2). Although it is likely that these structural changes in loop D are due to the substitutions in the IDCL, this conclusion cannot be stated definitively because the side chains of residues 43 and 44 in loop D of the mutant protein structure are located near a symmetry related PCNA molecule in the R3 space group.

It is interesting to note that these structural changes in the L126A/I128A mutant protein alter the ability of PCNA to bind small molecule ligands on its front face. In the structure of the L126A/I128A mutant protein, we observe clear electron density in the vicinity of the IDCL, loop B, and loop D into which we modeled a portion of a PEG molecule and a chloride ion from the crystallization conditions (Fig 5C). To our knowledge, binding of small molecule ligands at this site has not previously been observed with PCNA. This is strong evidence that the ability of the front face of PCNA to bind ligands has been altered in this mutant protein.

### 3.5. Impact of all three double alanine substitutions on the surface structure of PCNA

**Fig 6:**
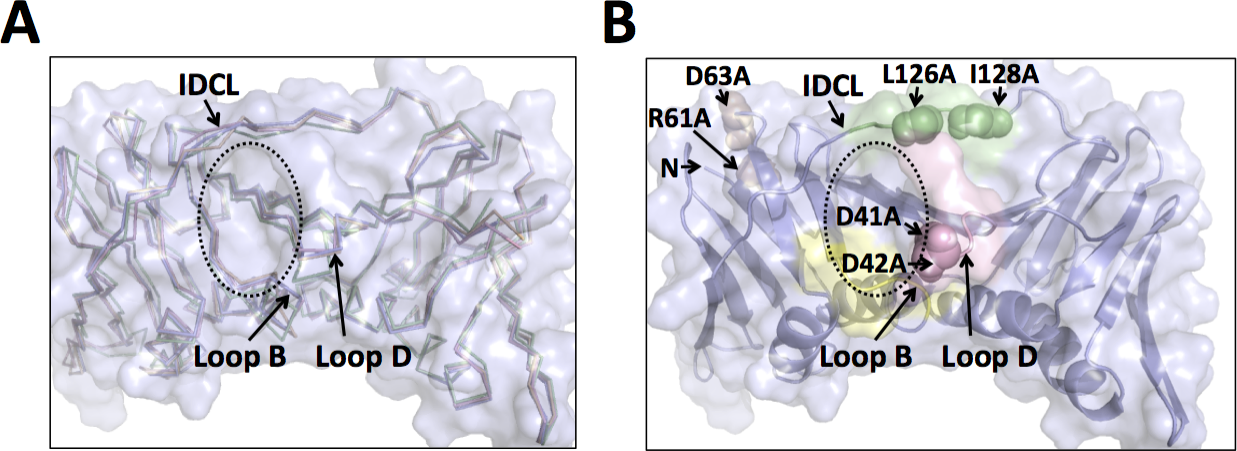
Comparison of the surface cavity in the wild-type and mutant PCNA proteins. (A) Close up of an overlay of the wild-type PCNA protein (light blue), the D41A/D42A mutant PCNA protein (light pink), the R61A/D63A mutant PCNA protein (wheat), and the L126A/I128A mutant PCNA protein (pale green) are shown in the ribbon representation. The positions of loop B, loop D, and the IDCL are indicated. The edge of the surface cavity is highlighted with a dashed ellipse. (B) Close up of the structure of the wild-type PCNA protein (light blue) is shown in the cartoon representation. The locations of residues D41 and D42 (light pink), residues R61 and D63 (wheat), and residues L126 and I128 (pale green) are shown in the sphere representation.

Loop B, loop D, and the IDCL form the edges of a prominent surface cavity on the front face of wild-type PCNA (Fig 6). Remarkably, this cavity is modified in all three mutant PCNA proteins, even though the three double alanine substitutions are located in quite different regions of the PCNA monomer. One of the three PCNA mutations, D41A/D42A, is located directly within one of the loops that defines this cavity. The I126A/L128A and R61A/D63A mutation sites, however, are approximately 15 Å and 30 Å away from the center of this cavity, respectively.

In the structure of the D41A/D42A mutant PCNA protein, the replacement of the larger, negatively charged aspartate residues with smaller, non-polar alanine residues causes alterations (>1 Å) in the positions of the backbone of several amino acid side chains that line the edges of the surface cavity, including residues 41 and 43 in loop D and residue 123 in the IDCL. Together, these changes result in the formation of a smaller, more “closed” surface cavity compared to the cavity in the wild-type structure. The overall shape of this cavity is similarly altered in the R61A/D63A mutant protein structure. Here, there are alterations (>1 Å) in the backbone of several amino acid residues along the edges of the cavity, including residues 21 and 22 in loop B and residues 123 and 124 in the IDCL. Again, these changes result in a smaller cavity compared to the wild-type structure. Of the three mutant proteins crystallized, the L126A/I128A substitutions cause the most dramatic changes to this surface cavity compared to the wild-type protein. Here, there are alterations (>1 Å) in the backbone of many amino acid residues along the edges of the cavity, including residues 21 to 24 in loop B, residues 41 to 44 in loop D, and residues 123 to 129 in the IDCL. As noted above, the structural changes observed with this mutant protein are sufficient to alter the ability of PCNA to bind ligands in this surface cavity.

## 4. Discussion

A number of separation-of-function mutations in PCNA have been identified in yeast that cause defects in one or more of these DNA-templated processes. The X-ray crystal structures of many of these mutant PCNA proteins have been determined to identify the structural implications of the amino acid substitutions and how they lead to these defects. For instance, over sixteen PCNA mutations have been identified that partially or completely block translesion synthesis (TLS), the process by which DNA polymerases replicate through damaged templates [35,44,46]. All of these mutation sites are clustered together within the PCNA subunit-subunit interface that forms the homotrimer. The X-ray crystal structures of PCNA containing two of these amino acid substitutions, E113G and G178S, show that these mutations cause substantial disruptions of the subunit interface itself. Similarly, over fourteen PCNA mutants have been identified that disrupt mismatch repair (MMR) to varying extents *in vivo* [48]. These amino acids are clustered at three distinct sites on the PCNA ring, most of which are located at the trimer interface or near the C22 residue on loop B. The X-ray crystal structures have been determined of two of these mutant PCNA proteins, C22Y and C81R [45]. Unlike the substitutions that block TLS, these two mutations are widely separated in space within the PCNA subunit and cause two distinct structural alterations in PCNA near each respective mutation site.

In this study, we focused on three separation-of-function amino acid substitutions in PCNA that cause defective gene silencing and exhibit reduced CAF-1 association with chromatin *in vivo*, as well as inhibit binding to CAF-1 *in vitro* partially or completely (D41A/D42A, R61A/D63A, and L126A/I128A) [25]. Similar to the MMR-defective mutant PCNA proteins, the substitutions studied here are located in three distinct and widely separated positions within the PCNA ring. Interestingly, however, these three substitutions all induce structural changes within the same region within PCNA and therefore appear to function through a common structural basis, which is dissimilar to what was observed with the MMR-defective mutants. Localized disruptions like these that are caused by distal mutations have not been previously observed with PCNA mutations. This finding demonstrates that, without structural data, one cannot assume that the specific location of an amino acid substitution within PCNA is the major region affected by that mutation. In addition, this type of structural mechanism highlights the complexity of the overall quaternary structure of PCNA and the vast array of functions that it regulates.

Most PCNA-interacting proteins contact PCNA via a conserved PIP motif, which interacts with PCNA in a PIP-interacting region that is located between the two domains of PCNA on a single subunit near the IDCL. Because the Cac1 subunit of CAF-1 contains a PIP motif, it is likely that CAF-1 binds PCNA at this region. However, because the three PCNA amino acid substitutions discussed here are defective in CAF-1 binding and do not affect DNA replication, CAF-1 must make additional contacts with PCNA outside of the canonical PIP-interacting region. Secondary contacts have been observed between PCNA and other PCNA-binding proteins, including FEN1, p21, RFC, and the Cdc9 DNA ligase [49–52]. In addition to the canonical PIP-PCNA interaction, all of these proteins contain regions flanking their PIP motifs that bind to PCNA at the IDCL, the C-terminus, or both [33]. Our structural data show that all three of the non-CAF-1-binding mutant PCNA proteins have perturbations at a common surface cavity that is approximately 10 Å away from the canonical PIP-interacting region. Together, these results suggest that the surface cavity is a novel secondary interaction site on PCNA that is required for its interaction with CAF-1.

It is important to note that the structural results presented here do not provide definitive proof that this surface cavity on PCNA is a novel secondary interaction site for binding CAF-1. First, it is possible that the phenotypes of cells producing these mutant PCNA proteins may not be entirely due to a defect in CAF-1 binding. While the D41A/D42A and L126A/I128A mutant proteins are completely defective in CAF-1 binding, the R61A/D63A mutant is only partially defective in CAF-1 binding [25]. This suggests that the defect in gene silencing may be due to the inability of the R61A/D63A PCNA mutant protein to stimulate the catalytic activity of CAF-1. This is a common mechanism by which separation of function PCNA mutant proteins block pathways such as TLS and MMR [35,44–46]. This cannot be ruled out as a possibly. Nevertheless, the R61A/D63A mutant protein still does not bind CAF-1 with the same affinity as does wild-type PCNA and this supports the model that this cavity is a binding site for CAF-1. Second, it is possible that the three mutations in PCNA disrupt its interaction with another factor that is required to stabilize the CAF-1-PCNA complex. We believe that this is unlikely, however, as studies demonstrating reduced binding between these mutant PCNA proteins and CAF-1 were performed *in vitro* using purified proteins [25]. Therefore, binding was not affected by the presence of other PCNA-interacting proteins, supporting the hypothesis that the cavity is a CAF-1 binding site. Third, it is possible that CAF-1 binds to one or more distinct sites elsewhere on the surface of PCNA. However, we believe that this is unlikely because these PCNA mutant proteins, which completely or partially disrupt interactions between PCNA and CAF-1, do not have significant or obvious structural changes in other regions of PCNA. Furthermore, the structural changes in this surface cavity induced by these three distinct sets of amino acid substitutions collectively provide strong support that this cavity is a binding site for CAF-1.

If this surface cavity identified in PCNA is a novel binding site for CAF-1, then it is probable that other proteins contact PCNA in the same manner. For instance, two strong candidates include the histone chaperones that are involved in CAF-1-independent nucleosome assembly, Hir1 and Asf1. Genetic studies found that gene silencing in cells containing PCNA with double alanine substitutions at either L126/I128 or D41/D42 were not significantly reduced further by the deletion of either Hir1 or Asf1 [27], suggesting that these PCNA mutants disrupt silencing by both CAF-1-dependent and CAF-1-independent pathways. Although further structural studies are required for a full understanding of the mechanism of binding between PCNA and CAF-1, Hir1, or Asf1, this unique secondary interaction likely plays an important role during nucleosome assembly and may confer a high affinity interaction with CAF-1 or other histone chaperone proteins. This would allow PCNA to discriminate between its multiple binding partners and would grant CAF-1 and potentially other histone chaperones access to PCNA during DNA replication.
